# Development of epimedin A complex drugs for treating the osteoporosis

**DOI:** 10.1007/s10856-020-06472-9

**Published:** 2021-01-27

**Authors:** Ying Liu, Yanan Bi, Lijuan Chai, Lei Song, Juyang Huang, Qin Wang, Yunzhang Li, Kun Zhou

**Affiliations:** 1 Institute of Traditional Chinese Medicine, Tianjin University of Traditional Chinese Medicine, Tianjin, 301617 China; 2 College of Veterinary Medicine, Inner Mongolia Agricultural University, Hohhot, 010018 China; 3 Tianjin State Key Laboratory of Modern Chinese Medicine, Tianjin, 301617 China; 4 Key Laboratory of Pharmacology of Traditional Chinese Medical Formulae, Ministry of Education, Tianjin, 301617 China; 5 Tianjin Key Laboratory of Chinese Medicine Pharmacology, Tianjin University of Traditional Chinese Medicine, Tianjin, 301617 China; 6 School of Integrative Medicine, Tianjin University of Traditional Chinese Medicine, Tianjin, 301617 China

## Abstract

Osteoporosis is the most common disease involving bone degeneration. As the age of the population increases, the prevalence of the disease is expected to rise. However, current treatment methods do not provide a desirable solution for the restoration of the function of degenerated bones in patients with osteoporosis. This led to emergence of controlled delivery systems to increase drug bioavailability and efficacy specifically at the bone regeneration. In this study, an epimedin A (EA) complex drug system was prepared by solution blending method. In vitro cell-based experiments showed that the EA complex drug could significantly promote the differentiation and proliferation of osteoblasts and increase the alkaline phosphatase activity, calcium nodule formation, and the expression of osteogenesis-related genes and proteins. In vivo experiments further demonstrated that this novel drugs remarkably enhanced bone regeneration. These results suggest that EA may be used for the treatment of osteoporosis.

## Introduction

Osteoporosis is a degenerative bone disease, which is commonly related to aging, and manifests itself as a decrease in bone mass and valley, leading to secondary fractures [1–3]. Conventional methods of treating osteoporosis are limited to the administration of multiple drugs to induce bone formation, effectively preserve bone mass, and increase bone strength [4–7]. With the deepening of research on osteoporosis, the related therapeutic drugs for osteoporosis have progressed from the early basic drug for bone mineral supplementation to the current stimulating bone formation. These drugs can inhibit osteoclast differentiation and reducing bone resorption, and improve bone microstructure, enhance bone density, and reduce fracture risk. However, the drug utilization rate is low, and long-term use of drugs results in drug resistance and adverse side effects in the body. The emergence of controlled delivery systems using biomaterial scaffolds has shown the prospect for developing local long-term treatments for osteoporosis patients [8–12]. For example, Mora-Raimundo et al. [13] designed novel drug-loaded nanoparticles, which showed the ability to inhibit osteoporosis-related genes and promote osteogenic marker expression in the treatment of osteoporosis. Massaro et al. [14] developed a simvastatin/nanofibrillated cellulose sustained-release system, which showed a good capacity for the promotion of bone formation.

*Epimedium* is a traditional Chinese medicine that alleviates the symptoms of kidney yang deficiency, strengthens muscles and tendons, and attenuates rheumatism. The recent study found that epimedin drug can be effectively improve the culture of stromal cytokines in the body, induce the bone marrow stromal cells to produce stem cell factor, and promote cell metabolism and protein synthesis, among them are significant for cell proliferation and osteoblast differentiation [15, 16]. Meanwhile, epimedin A (EA) drugs have been proved as an effective strategy that osteoclasts and osteoblasts can achieve functional correspondence, therapy effectively promoting bone resorption by the expansion of osteoporosis, and reduction of osteoclast absorption [17]. Especially, EA is one of its active ingredients, with a relatively high content in *Epimedium* [18, 19]. According to previous reports, EA shows an excellent efficacy against senile osteoporosis [19]. In addition, the gel system is also a good drug delivery vehicle [20–22]. A simple method is applied to inject bioactive drugs into the injectable gel system, and then, the hydrogel network loaded with various drug is implanted into the bone defect site using a syringe to achieve the drug treatment effect. This controlled drug release scaffold can reduce the frequency of medications and improve the drug absorption.

In this study, we constructed a thiolated gellan gum (TGG) loaded with EA complex drug system. A systematic study of the cell activity and in situ sustained-release behavior of this system, as well as its ability to promote osteogenic differentiation, was conducted in vitro. Furthermore, a castrated mouse model was constructed to evaluate the potential of the EA drug for the treatment of osteoporosis in vivo.

## Materials and methods

### Drugs and reagents

EA was purchased from Chengdu Pufei De Biotech Co., Ltd. Chemicals including glycerol 2-phosphate disodium salt hydrate (β-GP), ascorbic acid, alizarin red, cetylpyridinium chloride (CPC), and Trizol were purchased from Sigma. Estradiol valerate tablet (Progynova) is a product of DELPHARM Lille S.A.S (France). Alpha-minimal essential medium was purchased from Hyclone (Logan, UT, USA). Fetal bovine serum (FBS) was obtained from Biological Industries (Kibbutz Beit Haemek, Israel). All other biochemical reagent were of analytical grade and used as received.

### Preparation of TGG/EA complex

Firstly, TGG was modified by previous method [23]. The prepared TGG (1.5 g) was dissolved in deionized water (10 mL) at 60 °C, the obtained pregel solution was injected into the specific mold, and complete a sol–gel transition at 37 °C condition to produced injectable TGG hydrogel. Moreover, the EA was dissolved into phosphate-buffered saline (pH 7.4, 0.1 M) solution and evenly mixed with TGG solution to prepared the loaded-EA (2, 1, and 0.5 mg/mL) TGG hydrogels, which was named as H, M, and L loaded-EA hydrogel group, respectively. All prepared samples are sterilized for further used.

### In vitro cell culture

Mouse preosteoblastic cell line (MC3T3-E1) was used to study the cell growth behavior of TGG/EA complex drug. Prior to cell seeding, the resulting samples (10 mm in diameter and 1 mm in thickness) were soaked in 75% alcohol for 30 min and irradiated for 2 h under UV light for sterilization, and then the sterilized samples were immersed in culture medium for 2 h. The cells were seeded on the TGG/EA complex scaffolds (2 × 10^4^ cells/well in 24-well plates), and then incubated in 37 °C humidified condition with 5% CO_2_ atmosphere for 14 days, the culture medium was changed every 2 days. Meanwhile, cells seeded directly on 24-well cell culture plates and cultured with same amount of EA at the same density were served as control groups. After 3, 7, and 14 days’ culture with Minimum Eagle’s Medium (MEM-α, Invitrogen) supplemented with 10% FBS and 1% penicillin, MC3T3-E1 proliferation was measured by cell counting kit-8 (CCK-8, Jiancheng Co., Nanjing, China). The cell proliferation results are showed as optical density assessed at 450 nm.

Morphology of extracellular protein (bone morphogenetic protein-2 (BMP-2) and actin) immunofluorescence staining was performed according to previous literature reports for preliminarily evaluating the osteogenesis performance [18, 24]. After cultured for 5 days, the cells were immunostained by rabbit monoclonal antibody (ab73412, Abcam, USA) and goat anti-rabbit IgG secondary antibody (SA00009-2, Proteintech Group). Then, each specimen was immunostained with rhodamine-conjugated phalloidin (No. R415, Life Technologies, USA) for 40 min and with DAPI for 15 min in the dark to target actin filaments and nuclei, respectively. Specimens were fixed on microscopic slides, and confocal laser scanning microscopy was then performed.

### Quantitative and qualitative analysis of alkaline phosphatase (ALP) and calcium deposition

In vitro osteogenic induction was carried out using osteogenic medium (Cyagen Biosciences, Inc., USA). MC3T3-E1 were cultured on TGG, EA, and TGG/EA samples with osteogenic medium served as experimental group, and cultivated in normal medium as a control. After the cells were cultured for 14 days, the ALP kit and the bicinchoninic acid protein kit was applied to estimate the effect of the TGG gels loaded with EA on the early differentiation of MC3T3-E1 cells, and the qualitative analysis of ALP activity was recorded using a stereomicroscope (Stemi 2000-C, Carl Zeiss, Germany). Moreover, the calcium deposition of the cells was qualitatively analyzed by adding 1% alizarin red stain. The quantitative analysis by adding a 10% CPC solution and the absorbance was measured at 562 nm. Both the results of ALP stained and alizarin red stained were observed by the stereomicroscope.

### Western blot assays

The expression of osteogenesis protein including actin, osteoprotegerin (OPG), receptor activator of NF-κB ligand (RANKL), phospho-extracellular signal-regulated kinase (P-ERK), and extracellular signal-regulated kinase (ERK) of MC3T3-E1 cells cultured on TGG/EA hydrogels with different loadings (0.5, 1, and 2 mg/mL were abbreviated as L, M, and H, respectively) was evaluated by western blot. After 14 days of cell culture, cells were lysed by the addition of cold radioimmunoprecipitation assay lysis buffer (Beyotime, China) and equal amounts of protein (10 µg) were subjected to SDS-PAGE gel electrophoresis. Then, the proteins were transferred onto polyvinylidene difluoride membranes. After 2 h blocking at room temperature in 5% skimmed milk, the membranes were incubated with actin, OPG, RANKL, P-ERK, and ERK primary antibodies overnight, followed by incubation with an appropriate secondary antibody conjugated to horseradish peroxidase. The blots were visualized by enhanced chemiluminescence using an ECL kit (Amersham Pharmacia Biotech). Signals were detected using a VersaDoc™ Imaging System (Bio-Rad). The β-actin antibody was selected as the internal control. And three replicates were used for every sample testing.

### Animals and treatment

Young mature virgin female mice (Beijing HFK Bioscience Technology Co.) that had sham operations (control) or underwent bilateral ovariectomy (OVX) of 8 weeks of age were used. After 6 weeks, the survived mice were randomly divided into five groups: model group, positive drug group (with estradiol valerate), and low, medium, and high EA concentration treated groups (5, 10, and 20 mg/kg, respectively), each group had 12 mice. Medicines were administrated intragastrically once a day for 8 weeks. Mice in control group and model group were treated with water. Finally, the mice were weighted and sacrificed. Uterus was weighted to detect uterus index, tibias were used to detect bone density and strength, and femurs were used to analyze pathology and extract RNA.

#### Bone strength analysis

Right femur was used to test bone strength in mode 1 using YLS-16A small animal bone strength analyzer (Jinan Yiyan Technology Co. Ltd., Jinan, China), and the result is the femur maximum load capacity which indicate the maximum force being applied to the femur until it was fractured.

#### Bone density analysis by micro-computed tomography (CT)

Right tibia was preserved in 10% methanal. Bone structural indices and trabecular bone morphometry were performed using viva CT40 (SCANCO Medical AG, Zurich, Switzerland). Meanwhile, the number of connections of bone (Conn.D), degree of anisotropy (DA) was the ratio of longest vector of mean intercept length (MIL) sensor over shortest vector of MIL sensor, relative bone volume/total volume (BV/TV), trabecular bone number (Tb.N), trabecular thickness (Tb.Th), and trabecular bone separation (Tb.Sp) were calculated by plate model, and the scan region was below the epiphyseal growth plate of proximal tibia, extending about 1 mm toward the distal direction.

#### Histomorphological analysis

Left tibia was fixed with 10% buffered formalin and decalcified with 10% EDTA for 1 month, then embedded in paraffin to be sliced at 5 µm. Sections were stained with hematoxylin and eosin (H&E). Histomorphological examination was performed by BX51 optical microscope (Olypums, Tokyo, Japan).

#### RNA extraction and quantification by real-time PCR

Total RNAs were isolated using Trizol reagent (Thermo Fisher Scientific, Waltham, MA, USA). Reverse transcription was performed with 1 µg of RNA using the All-in-one First-strand cDNA Synthesis SuperMix (Transgen, Beijing, China). Quantitative real-time PCR analysis was performed by the addition cDNA and SYBR green master mix in micro-AMP optical tubes using the CFX96 Real-Time PCR Detection System (Bio-Rad, USA). The expression of genes relative to that of GAPDH was determined by the 2^−ΔΔCt^ method [25]. The primer sequences for the real-time RT-PCR analysis are listed in Table 1.

**Table 1:** Primer sequences used for RT-PCR

Gene	Primer sequence (5′~3′)
IBSP-F; R	TGTCCTTCTGAACGGGTTTC; TCGTTGCCTGTTTGTTCGTA
Runx2-F; R	GACGAGGCAAGAGTTTCACC; GTCTGTGCCTTCTTGGTTCC
ATF4-F; R	CCGAGATGAGCTTCCTGAAC; TTGGCCACCTCCAGATAGTC
OPN-F; R	CACCATTCGGATGAGTCTGA; CCTCAGTCCATAAGCCAAGC
GAPDH-F; R	AGGTCGGTGTGAACGGATTTG; TGTAGACCATGTAGTTGAGGTCA

All animals experiments were approved by the Laboratory Animal Ethics Committee of Tianjin University of Traditional Chinese Medicine (Permit number: TCM-LAEC 2015003).

### Statistical analysis

All experimental results were expressed as the mean ± standard deviation, and an analysis of variance test was performed for statistical analysis. When *p* < 0.05, it means that the difference is significant (*), whereas *p* < 0.01 indicates that the difference is very significant (**), which are compared to the control group.

## Results and discussion

### In vitro cell proliferation, drug release results, and cell staining results

**Fig. 1:**
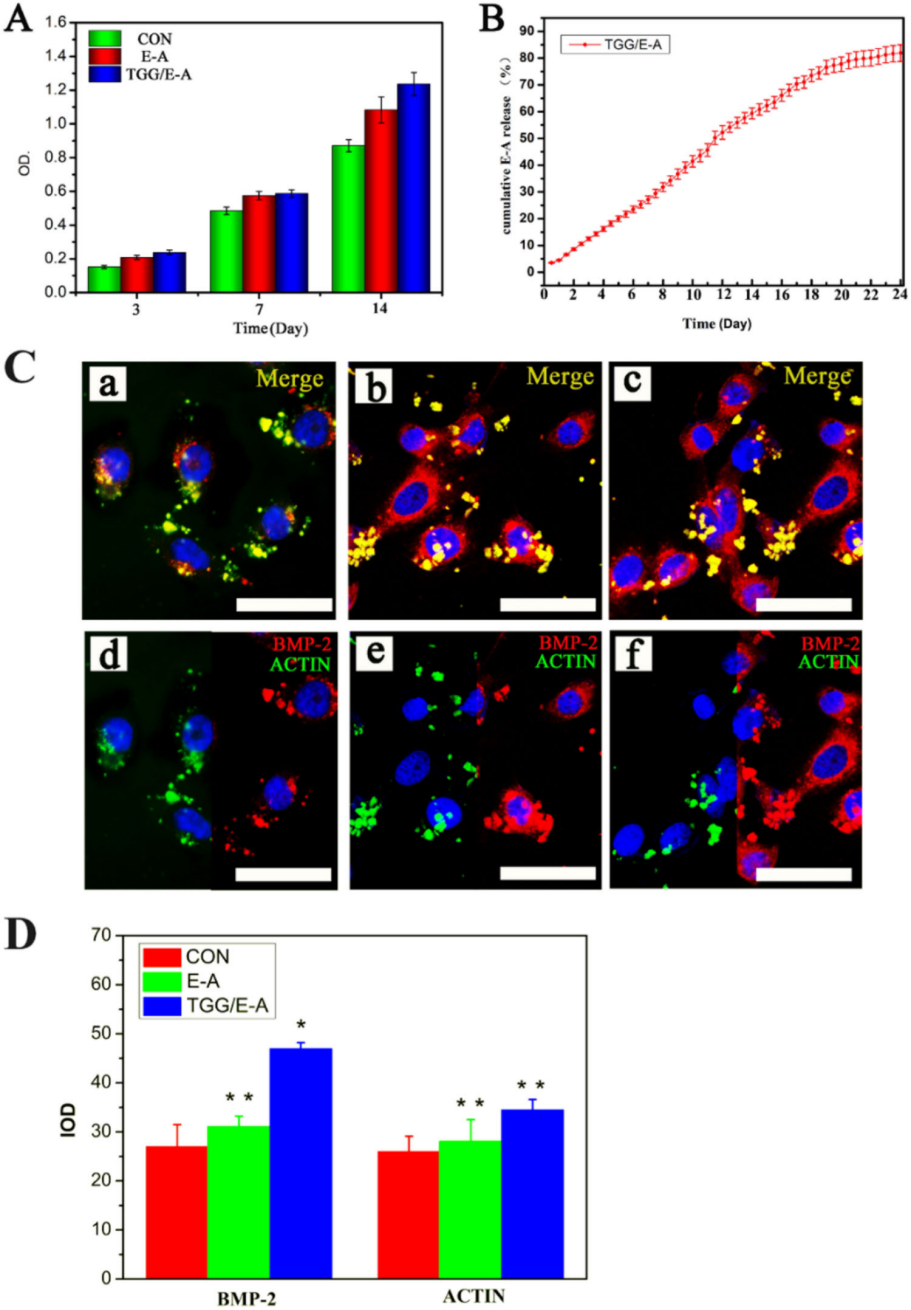
**A** Results of the CCK-8 assay of MC3T3-E1 cells after 14 days of culture. **B** EA release curves from TGG. **C** Images of BMP-2 and actin fluorescence staining in MC3T3-E1 cells cultured on (a, d) a blank orifice plate, (b, e) with EA, and (c, f) with TGG/EA for 5 days. Scale bar = 50 µm. **D** Quantitative statistics of the mean fluorescence densities of BMP-2 and actin in the fluorescent staining images

The results of materials (Fig. S1) indicate that TGG was successfully prepared and regarded as a promising drug carrier. Firstly, the CCK-8 was used to measure the cell viability. Figure 1A shows the proliferation of MC3T3-E1 cells cultured in vitro for 14 days. The results showed that the cell proliferation increased as the culture time was prolonged. The experimental groups with EA showed higher levels of proliferation than did the blank control group after 3, 7, and 14 days of culture, which may be due to the promotion of cell proliferation by the drug, and the TGG/EA group showed the highest proliferation. Figure 1B shows an in vitro EA release curve from TGG. EA showed no significant burst release and could maintain a sustained release for nearly 24 days. This result was probably due to the interaction between the thiol groups in the TGG chain and the EA drug, resulting in the stability of EA in the gel network structure [26, 27]. This long-term and sustained release may be beneficial for the regeneration and repair in tissue engineering. The mechanism of sustained drug release can allow avoiding repeated administration and improve the absorption rate of the drug.

BMP-2 is a transforming growth factor that promotes the differentiation and maturation of osteoblasts [18, 19]. BMP-2 participates in the process of bone and cartilage growth, development, and remodeling and accelerates the repair of bone defects [28–30]. Therefore, BMP-2 can be used to initially evaluate the osteogenic capacity of MC3T3-E1 cells. In this experiment, double immunofluorescence staining was used to specifically label BMP-2 (red) and actin (green), which were quantified and qualified to analyze pro-osteogenesis effects (Fig. 1C) on MC3T3-E1 cells in the three groups, with quantitative statistical analysis data shown in Fig. 1D. Both expression levels of BMP-2 and actin in the EA and TGG/EA group were significantly higher than blank control group, which may be attributed to the promotion of osteogenesis in MC3T3-E1 cells by the EA drug. The expression levels of BMP-2 and actin in the groups supplemented with EA were 29.4 and 47.6% higher than those in the blank control group, respectively, further revealing the pro-osteogenic effect of EA.

### Evaluation of osteogenic performance

**Fig. 2:**
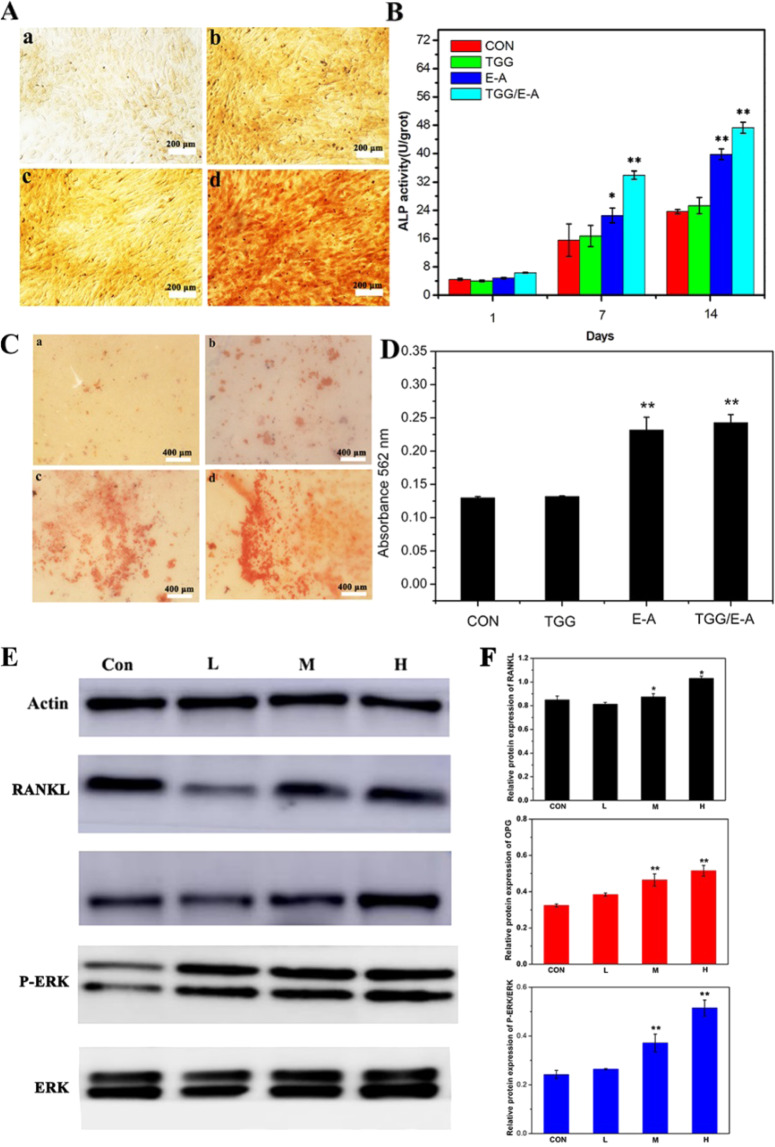
**A** ALP staining and **B** ALP activity of MC3T3-E1 cells after culturing on (a) a blank orifice plate and that with (b) TGG, (c) EA, and (d) TGG/EA for 14 days. **C** The corresponding results of alizarin red staining (ARS) and **D** its quantitative analysis. **E** Western blot results of related protein expression of MC3T3-E1 cells after culturing on TGG, TGG loaded with low (L), medium (M), and high (H) EA concentrations. **F** The corresponding results from quantitative analysis

ALP and alizarin red staining were used to further validate the TGG/EA complex system as a platform for effective osteoinduction. ALP is an enzyme that catalyzes the hydrolysis of phosphate esters at alkaline pH and can be used as an early marker in the process of osteogenic differentiation. ALP staining of MC3T3-E1 cells was performed on day 14, and ALP in the cells was stained brown (Fig. 2A). Compared with the control group, the drug groups secreted more ALP, which was further confirmed by the quantitative analysis of the ALP activity (Fig. 2B). On days 1, 7, and 14, the ALP expression in the EA and TGG/EA groups was higher than that in the control group, indicating that the presence of EA significantly induced the ALP secretion. In addition, calcium deposition was used as a late marker in the process of osteogenic differentiation, and the formation of mineralized nodules of osteoblasts was detected using alizarin red staining [31–33]. As shown in Fig. 2C, D, compared with the control, the TGG group formed no more mineralized nodules after osteoinduction. However, the formation of mineralized nodules was significantly higher in the EA containing groups, especially in the TGG/EA group, where numerous mineralized nodules could clearly be observed on day 14. These results indicate that EA can promote osteogenic differentiation of cells and calcium deposition by stimulating gene expression and secretion of osteogenic markers [2, 9, 34].

To further explore the effects of EA on osteogenic differentiation, the expression levels of OPG, RANKL, and ERK were quantitatively analyzed by western blotting. As shown in Fig. 2E, F, the expression of OPG and P-ERK in the blank control group was significantly lower than that in the TGG/EA complex groups, and the expression of the osteogenesis-related protein increased with the increase of the EA drug loaded in the TGG/EA complex system. Compared to the CON group, RANKL, OPG, and ERK expression by MC3T3-E1 cells cultured on M and H group was slightly increased to different degrees, and the highest expression level of the osteogenesis-related protein was observed in the H group. Thus, the introduction of the EA drug showed a significant promoting effect on the expression of osteogenesis-related proteins.

### Evaluation of bone-promoting properties in vivo

**Fig. 3:**
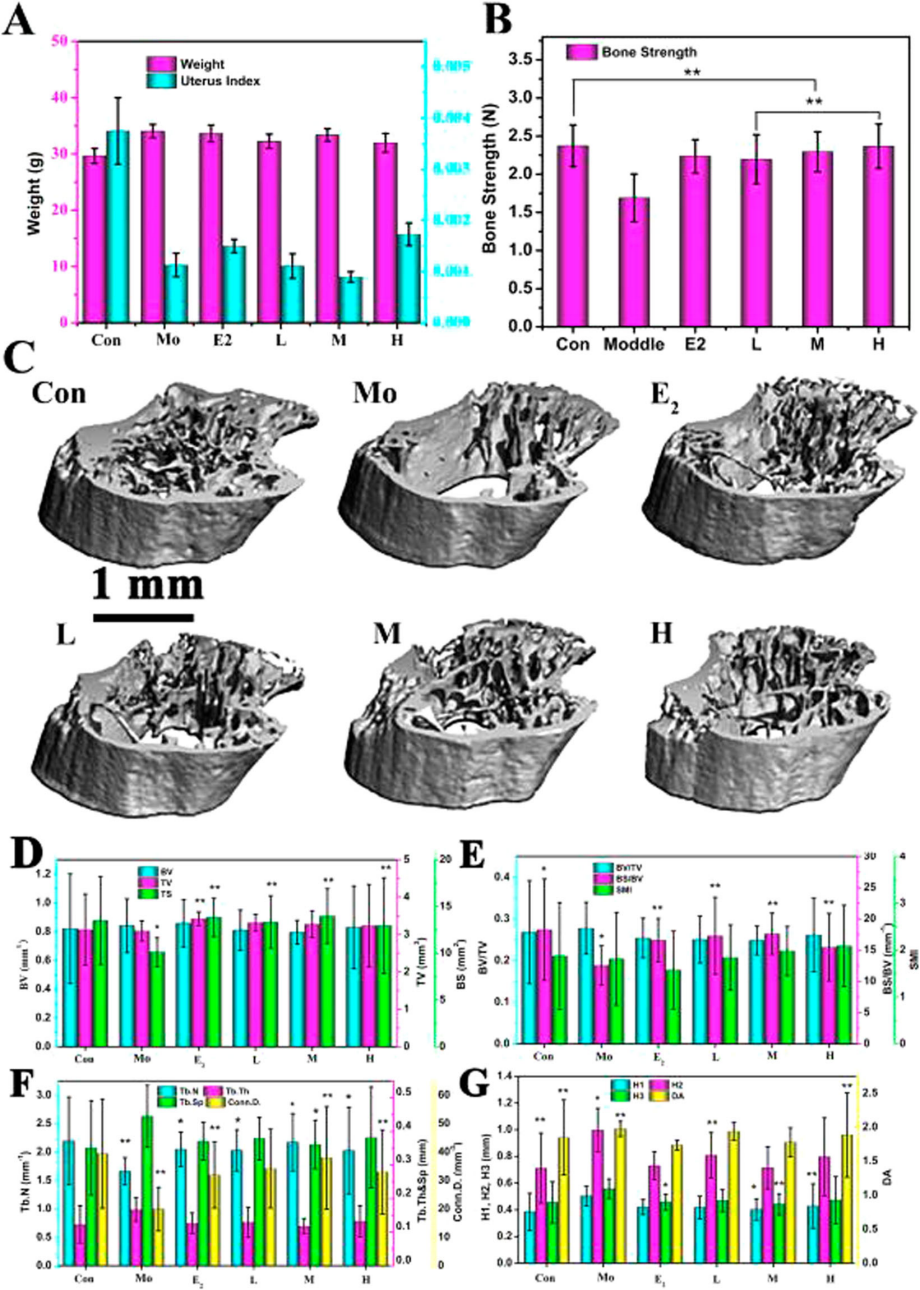
**A** Weight and uterus index of the control, model, and castrated mice after 8 weeks of treatment with estradiol valerate (E_2_) and with EA different concentrations (L, M, and H). **B** Bone strength and **C** bone computed tomography images of the corresponding samples. **E**–**H** The effects of treatments with E_2_ and EA (L, M, and H concentration) on the three-dimensional structure of the mouse tibia, including BV, TV, BS, BV/TV, BS/BV, SMI, Conn.D, Tb.N, Tb.Th, Tb.Sp, H1, H2, H3, and DA

On account of its good osteogenic performance in vitro, we constructed a bone osteoporosis model using castrated mice aiming to further investigate the therapeutic effect of EA drugs on osteoporosis in vivo. For 8 weeks after castration, the mice were administered different treatments. Mice treated with EA drugs were used as experimental groups, untreated mice were used as a model control group, and those treated with E_2_ were used as a positive control group. Successful establishment of an osteoporosis model often requires evaluation of multiple aspects, namely, changes in the bone mass, mechanical properties, bone microstructure, and biochemical metabolic indicators [35, 36]. In this experiment, the body weight, uterus index, bone strength, and bone microstructure were selected as observation indicators. Figure 3A shows the body weight and uterus index after administration of the EA drug. Compared with the blank control and model control groups, the direct E_2_ administration and EA drug groups of castrated osteoporosis mice tended to show higher body weights and uterus indexes; however, the differences were not significant. Statistical analysis showed that the bone strength was significantly higher in the drug treatment groups than in the model group (Fig. 3B), and the highest bone strength was observed in H group, which almost close to the value of normal bone. In addition, a CT scan of the tibia was performed in the mice using a vivaCT 40 bone densitometer to further evaluate the effect of the drug concentration on the bone microstructure. In the four drug treatment groups, a newly formed trabecular bone of the tibia and a transitional trabecular bone at the bottom of the calcified zone were observed (Fig. 3C). In particular, in the group treated with the high concentration (H) of EA drug, the number and shape of the trabecular bone were almost identical to those in the normal control group, while the bone structure was slightly improved in the M group. In contrast, epiphyseal chondrocytes of the upper tibia were not aligned in the model group. Obviously, an increase in the drug concentration in the complex drug system can further improve the bone structure, which is related to the ability of EA to promote osteoblast differentiation.

Further, the microstructure of the trabecular bone was analyzed using micro-CT images of different layers, and spatial parameters of the trabecular bone were measured, including the BV, TV, bone connection number (Conn.D), structure model index (SMI), bone surface area (BS), Tb.N, Tb.Th, Tb.Sp, and various heterogeneity indicators (DAs), and the results are shown in Fig. 3D–G.

**Fig. 4:**
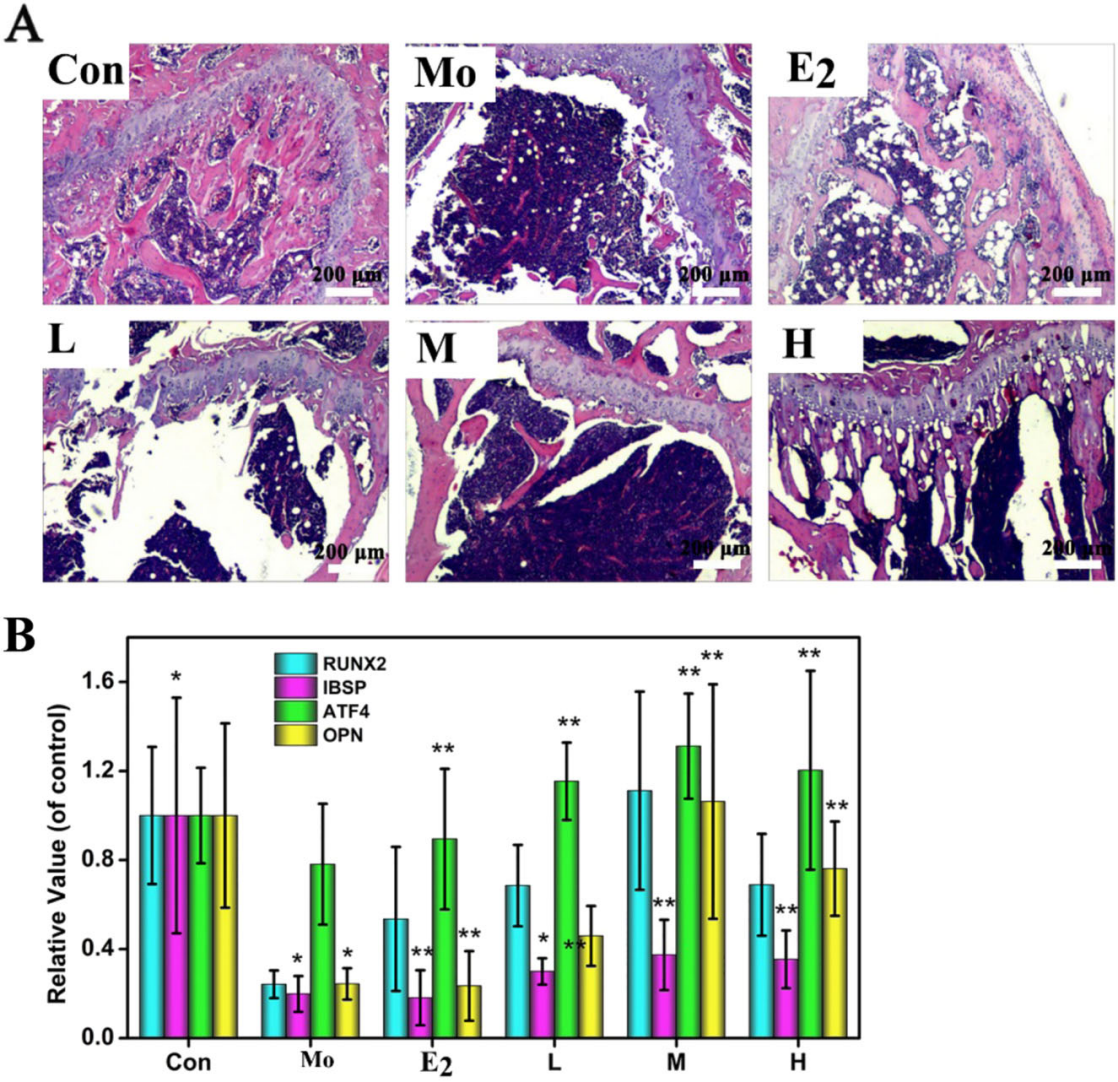
**A** Histomorphological photomicrographs of the tibia. **B** The corresponding PCR results (including RUNX2, IBSP, ATF4, and OPN) of the normal (Con), model, and experimental mice after 8 weeks of treatment with estradiol valerate (E_2_) and EA drugs with different concentrations (L, M, and H)

More severe osteoporosis results in smaller Conn.D values and larger Tb.Th and Tb.Sp values. In addition, the BV/TV ratio between the trabecular BV and the total BV is used as an important indicator to evaluate the effects of drugs on the bone mass. Antiosteoporosis drugs usually increase the percentage of the trabecular BV. In this study, Conn.D, TV, BS, and SMI values were significantly higher and DA values were significantly lower in the E_2_ and EA groups than in the model group. Particularly, OVX mice treated with higher loaded-EA hydrogel showed a more favorable serum marker pattern than even the positive control (estradiol valerate treated) mice; BV/TV, BS/BV value was higher in H group than in any other group, and BV/TV was significantly increased in both M and H group. This discrepancy may relate to sustained and effective release of antiosteoporosis EA drugs, and different results might have been obtained over a longer time course. These results indicated that EA exerted a good antiosteoporosis effect in castrated mice, and higher concentrations of the EA drug displayed stronger osteogenesis-promoting effects.

Histological assessment of the tibia was used to study the bone ingrowth TGG/EA hydrogel scaffold, and further verified the effect of TGG loading with EA concentrations on osteoinductive activity. As illustrated in H&E staining images (Fig. 4A), the model group almost did not show mature bone formation, only few new bone was found in the defect margins. A certain degree of osteoporosis is still found in E_2_ group, whereas in the experimental mice treated with the EA drug, the numbers and morphology of chondrocytes and the trabecular bone significantly improved compared with those in the E_2_ administration group. Especially, the H loaded EA groups found that a considerable amount of well-integrated bony tissue grew into the middle of the hydrogel scaffold, and epiphyseal chondrocytes of the upper tibia were aligned, the cell boundaries of the cartilage were clear, which was consistent with the CT scan images. In addition, the quantitative statistics of measurement of PCR showed that the RUNX2, IBSP, ATF4, and OPN values in the EA drug treatment groups were significantly higher than in the model group, the corresponding relevant value in the H group is close to those in the control group. These results indicated a good efficacy of controlled EA drug release for osteoporosis treatment using orally administered TGG hydrogels in castrated mice.

## Conclusions

In this study, TGG/EA complex drug was designed as a good controlled release system. The resulting system showed a good cell activity and promoted cell proliferation, differentiation, and spreading in in vitro experiments. Based on the in vitro drug release experiment, this system could effectively and sustainably release EA, which significantly promoted the MC3T3-E1 cell proliferation, ALP activity, and calcium nodule formation, as well as the expression of osteogenesis-related proteins. In addition, the PCR and micro-CT, H&E staining also were evaluated, which all demonstrated that the EA drugs with a high drug concentration could effectively increase the bone strength in castrated mice and significantly improve the bone microstructure. The findings further demonstrated a good bone remodeling effect of the EA drugs. Therefore, these results reveal the potential of EA drug as a novel approach to the treatment of osteoporosis.

## Supplementary information

Supplementary Information
