# Supplementary material for: Development of epimedin A complex drugs for treating the osteoporosis
Source: J Mater Sci Mater Med. 2021 Jan 27;32(1):17. doi: 10.1007/s10856-020-06472-9 (PMC7840628; doi:10.1007/s10856-020-06472-9)
Supplement: Supplementary file 1 — Supplementary Information [file 10856_2020_6472_MOESM1_ESM.docx]

**Supporting Information**

**Development of Epimedin A complex drugs for treating the Osteoporosis**

Ying LIU^1,2^, Yanan BI^1,3^,Lijuan CHAI^1,4^, Lei SONG^1,5^,Juyang HUANG^6^,Qin WANG^1,3^,Yunzhang LI^2,*^, Kun ZHOU^1,3,*^

1. Institute of Traditional Chinese Medicine, Tianjin University of Traditional Chinese Medicine, Tianjin 301617, China;

1. College of Veterinary Medicine, Inner Mongolia Agricultural University, Hohhot 010018, China;

3. Tianjin State Key Laboratory of Modern Chinese Medicine, Tianjin 301617, China;

4. Key Laboratory of Pharmacology of Traditional Chinese Medical Formulae , Ministry of Education, Tianjin 301617, China;

5. Tianjin Key Laboratory of Chinese medicine Pharmacology, Tianjin University of

Traditional Chinese Medicine, Tianjin 301617, China;

6.School of Integrative Medicine, Tianjin University of Traditional Chinese Medicine, Tianjin 301617, China.

*Correspondence should be addressed to Kun ZHOU, E-mail: [z.k.ken@263.net](mailto:z.k.ken@263.net), and Yunzhang LI E-mail: [liyunzhang1956@163.com](mailto:liyunzhang1956@163.com)

**Preparation and characterization of hydrogel materials**

Characterization: TGG was characterized by ^1^H NMR spectrum to exactly determine its chemical structure, and the measurement was performed on a Bruker 500 MHz Ascend. The morphology of hydrogels was observed using a scanning electron microscope (SEM, LEO1530 VP, Philips, Netherlands). Briefly, the freeze-dried gel specimens were cryogenically fractured under liquid nitrogen, and sputter-coated with gold for observation. The rheological measurements of hydrogels were performed using a rotational rheometer (DHR, TA Instruments, USA) with parallel plate (diameter of 20 mm and a gap of 1 mm). The strain amplitude sweep test (γ= 0.001%-1000%, ω= 6.28 rad/s) and dynamic oscillatory frequency sweep measurements (ω= 0.1-100 rad/s, γ=1%) at 37^o^C was studied the viscoelastic properties of TGG gels.

**Evaluation of drug release properties**

E-A release properties from TGG hydrogel network structure was investigated by a UV–vis spectrophotometer. The hydrogel samples (3 parallel samples per group) loaded with E-A (2 mg/mL gel) were immersed in 5 mL of PBS and the solution was placed under 120 rpm shaking at 37 °C for 24 days. At predetermined time points, 1 mL of medium was taken out for the measurement of the concentrations of EA, and the equal volume of fresh PBS was added. The amount of released EA was determined using a UV absorbance measure at 374 nm according to the PSI standard curve. The cumulative release of EA (%) was calculated with the equation: E-A(%) = (total release of EA/total load of EA in the sample) × 100%.

**
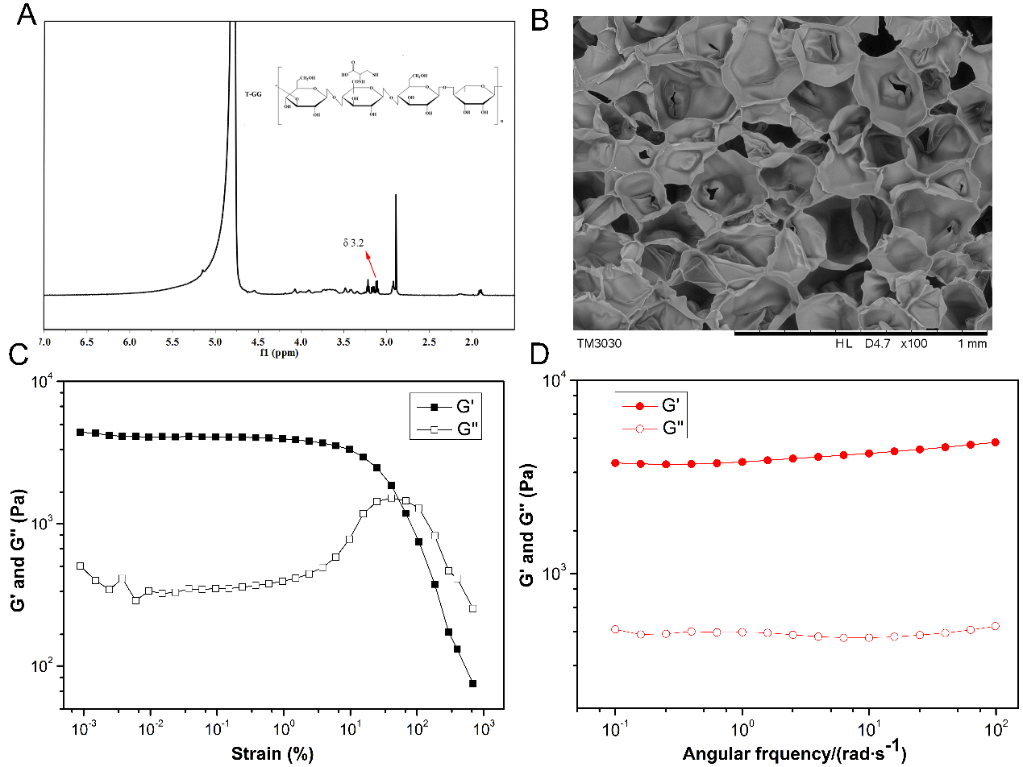
**

Fig. S1 (A) ^1^H NMR spectrum of modified TGG; (B) scanning electron microscopy image of TGG. The storage modulus (G) and the loss modulus (Gr) were plotted logarithmically versus the strain (C) and frequency (D) of hydrogel samples incubated at 37 °C.

GG was modified to TGG to improve its biological properties, and a ^1^H NMR spectrum of TGG is shown in Fig. 1A. A weak peak observed at 3.2 ppm was ascribed to -SH in the long chain, which confirmed that thiol groups were successfully incorporated in the molecule. Figure 2B shows a scanning electron microscopy image of the cryo-fractured morphology of TGG, exhibiting an open porous network structure and core interconnectivity, which is regarded beneficial for the transfer of nutrients to cells by a drug delivery vehicle [[23](#_ENREF_23)]. To investigate the viscoelastic behavior of TGG, rheological tests were carried out. The results of the strain amplitude sweep are shown in Fig. 1C, which displays a broad linear viscoelastic region and a great anti-shear ability, with the network merely collapsing at strain values over 80%, indicating a wide processing range and a shear-thinning (injectable) property. Frequency-dependent oscillatory shear rheology (Fig. 1D) of the corresponding hydrogel sample showed that the G′ and G″ values of the tested sample did not cross with an increase of the angular frequency, demonstrating the formation of a stable network structure of the hydrogel.
